# Adverse effects of nasopharyngeal swabs: Three-dimensional printed versus commercial swabs

**DOI:** 10.1017/ice.2020.297

**Published:** 2020-06-11

**Authors:** Kalpana Gupta, Pamela M. Bellino, Michael E. Charness

**Affiliations:** 1 VA Boston Healthcare System, West Roxbury, Massachusetts; 2 Boston University School of Medicine, Boston, Massachusetts; 3 Harvard Medical School, Boston, Massachusetts

*To the Editor—*To date, >6 million tests for COVID-19 have been performed in the United States, with the vast majority utilizing nasopharyngeal sampling.^1^ The need for large-scale testing in the COVID-19 pandemic has created a global shortage of commercial nasopharyngeal swabs. One approach to this shortage has been the 3-dimensional (3D) printing of nasopharyngeal swabs. Swabs printed on a 3D printer (3D swab) differ somewhat from commercially produced swabs: they having larger heads, less flexibility, and a plastic rather than cotton or polyester fiber tip. These 3D swabs are class 1 medical devices, and their diagnostic efficacy has been validated through field testing.^2^

Guidance on the safe collection of nasopharyngeal samples using commercial swabs is available in text and video format^3,4^; however, no data are available on the adverse effects of either commercial or 3D swabs, making it difficult to assess their relative safety. To expand testing at our medical center, we printed the Northwell prototype 3D swab using specifications obtained from the technology transfer office at the University of South Florida. As part of our safety assessment of this prototype, we identified adverse effects of NP swabbing in employees using both commercial and 3D swabs. Epistaxis occurred immediately or shortly following the removal of the swab in 5.0% of employees tested with the 3D swab and in 8.3% of employees tested with the commercial swab (Table 1). Epistaxis was usually mild and self-limited, although 1 employee required an emergency department visit for ongoing epistaxis after testing with a commercial swab. Other minor adverse effects included nasal discomfort, headache, earache, and rhinorrhea, which typically lasted hours to a day.

**Table 1.** Comparison of 3D Printed Nasopharyngeal Swabs Versus Commercial Swabs

Variable	Commercial Swab, No.	3D Swab, No.
Sample size	96	80
Epistaxis, no. (%)	8 (8.3)	4 (5.0)
Nasal discomfort	4	6
Headache	5	2
Ear discomfort	5	1
Rhinorrhea	5	1

Our finding that epistaxis is equally common after the use of 3D and commercial swabs provides reassurance that 3D swabs are a safe alternative to commercial swabs. However, the ~5%–10% incidence of epistaxis after nasal swabbing with either commercial or 3D swabs warrants caution in testing individuals at increased risk for bleeding. Nursing home residents have been disproportionately affected by COVID-19, and a recent point prevalence study of Medicare fee-for-service beneficiaries found that almost half of 37,787 nursing home residents were treated with oral anticoagulants.^5^ Rates of epistaxis after nasal swabbing should be studied in larger populations, including the elderly, and individuals at increased bleeding risk should be monitored after the procedure. Fortunately, less invasive methods of SARS-CoV-2 detection, such as midturbinate or saliva sampling, are on the horizon.
